# Supplementary material for: Microbial Co-Occurrence Patterns and Keystone Species in the Gut Microbial Community of Mice in Response to Stress and Chondroitin Sulfate Disaccharide
Source: Int J Mol Sci. 2019 Apr 30;20(9):2130. doi: 10.3390/ijms20092130 (PMC6539173; doi:10.3390/ijms20092130)
Supplement: Supplementary file 1 [file ijms-20-02130-s001.zip › ijms-475032-supplementary-final/Tables S1-S2 Figures S1-S4.docx]

**Supplementary materials**

**Table S1.** Topological properties of the global network inferred using a Random-Matrix theory (RMT) based network pipeline under various experimental conditions. N: healthy mice supplemented with PBS; C: healthy mice supplemented with a daily dose of 150 mg/kg bodyweight of chondroitin sulfate disaccharide (CSD) for 16 consecutive days; M: mice subjected to exhaustive exercise stress supplemented with PBS; S: the stressed mice supplemented with a daily dose of 150 mg/kg bodyweight of CSD for 16 days.

| **Network Indexes** | **N** | **C** | **M** | **S** |
| --- | --- | --- | --- | --- |
| Total nodes | 540 | 507 | 502 | 460 |
| Total links | 788 | 1278 | 782 | 854 |
| Total modules | 58 | 41 | 55 | 59 |
| Modularity | 0.842 | 0.797 | 0.897 | 0.808 |
| R square of power-law | 0.904 | 0.824 | 0.871 | 0.912 |
| Average degree (avgK) | 2.919 | 5.041 | 3.116 | 3.713 |
| Average clustering coefficient (avgCC) | 0.201 | 0.256 | 0.224 | 0.261 |
| Average path distance (GD) | 10.371 | 6.448 | 9.891 | 8.329 |
| Geodesic efficiency (E) | 0.132 | 0.198 | 0.144 | 0.173 |
| Harmonic geodesic distance (HD) | 7.578 | 5.060 | 6.933 | 5.774 |
| Maximal degree | 23 | 31 | 16 | 21 |
| Nodes with max degree | sp1742 | sp1835 | sp1844 | sp0350 |
| Centralization of degree (CD) | 0.037 | 0.052 | 0.026 | 0.038 |
| Maximal betweenness | 18727.51 | 19659.976 | 14941.842 | 18641.392 |
| Nodes with max betweenness | sp1291 | sp1559 | sp2143 | sp2019 |
| Centralization of betweenness (CB) | 0.12 | 0.147 | 0.113 | 0.17 |
| Maximal stress centrality | 160070 | 403109 | 127988 | 456940 |
| Nodes with max stress centrality | sp1674 | sp1042 | sp1903 | sp2019 |
| Centralization of stress centrality (CS) | 1.042 | 2.991 | 0.957 | 4.2 |
| Maximal eigenvector centrality | 0.374 | 0.251 | 0.361 | 0.319 |
| Nodes with max eigenvector centrality | sp1742 | sp1835 | sp1844 | sp0638 |
| Centralization of eigenvector centrality (CE) | 0.364 | 0.240 | 0.352 | 0.306 |
| Density (D) | 0.005 | 0.010 | 0.006 | 0.008 |
| Reciprocity | 1 | 1 | 1 | 1 |
| Transitivity (Trans) | 0.322 | 0.301 | 0.365 | 0.328 |
| Connectedness (Con) | 0.547 | 0.641 | 0.382 | 0.478 |
| Efficiency | 0.993 | 0.987 | 0.988 | 0.987 |
| Hierarchy | 0 | 0 | 0 | 0 |
| Lubness | 1 | 1 | 1 | 1 |

**Table S2.** Physiological parameters measured in mice. N: Healthy control mice + Phosphate-buffered saline or PBS; M: Exhaustive exercise stressed mice + PBS; C: Healthy mice supplemented with CSD at a daily dose of 150mg/kg body weight for 16 consecutive days; and S: Exhaustive exercise stressed mice supplemented with CSD at a daily dose of 150mg/kg body weight for 16 days. BUN: Blood urea nitrogen; CR: BUN to creatinine ratio. MDA: Malondialdehydes; and SOD: Superoxide dismutase.

| Animal_ID | SOD | MDA | BUN | CR |
| --- | --- | --- | --- | --- |
| N1 | 13.1478 | 4.2127 | 0.7992 | 3.9330 |
| N2 | 21.8738 | 0.5534 | 0.7191 | 2.9048 |
| N3 | 12.3272 | 6.2417 | 0.6430 | 3.1180 |
| N4 | 13.6749 | 2.2004 | 0.6717 | 3.3895 |
| N5 | 22.7955 | 1.9714 | 0.6574 | 3.8359 |
| N6 | 23.3146 | 1.6590 | 0.7144 | 3.5836 |
| N7 | 21.9965 | 6.4399 | 0.6374 | 3.7895 |
| N8 | 17.4457 | 0.4184 | 0.6774 | 3.1895 |
| N9 | 14.6527 | 1.2581 | 0.7008 | 3.4608 |
| M1 | 4.7340 | 6.8118 | 0.9368 | 4.0435 |
| M3 | 6.2314 | 6.6465 | 0.8784 | 4.0496 |
| M4 | 13.9674 | 10.0436 | 1.0116 | 4.2245 |
| M6 | 20.6318 | 7.1874 | 1.1782 | 4.3217 |
| M7 | 22.3991 | 9.1655 | 0.9751 | 3.9719 |
| M8 | 14.9565 | 6.8226 | 1.0706 | 4.2828 |
| M9 | 19.9463 | 7.3753 | 1.1066 | 4.0107 |
| M10 | 23.1091 | 7.3164 | 1.1362 | 4.2435 |
| C1 | 22.9125 | 1.3337 | 0.6478 | 3.2537 |
| C2 | 22.7105 | 7.2473 | 0.6478 | 3.6030 |
| C3 | 32.7505 | 1.2398 | 0.6812 | 3.4865 |
| C4 | 32.0320 | 2.3669 | 0.7239 | 3.6030 |
| C5 | 25.9165 | 0.3872 | 0.6621 | 3.5642 |
| C6 | 24.2995 | 1.6118 | 0.7049 | 3.5253 |
| S1 | 21.3365 | 4.3424 | 0.7286 | 4.1662 |
| S2 | 26.2932 | 3.6456 | 0.7191 | 3.3701 |
| S3 | 22.3132 | 3.9083 | 0.5711 | 3.0598 |
| S4 | 20.4552 | 6.5874 | 0.8645 | 3.0792 |
| S5 | 7.6945 | 4.9212 | 0.6764 | 3.1568 |
| S7 | 20.7334 | 5.9661 | 0.7097 | 3.7971 |
| S8 | 21.3181 | 4.4596 | 0.7195 | 3.4521 |

**Supplementary figures**

Visualization of global networks with modules identified using the fast greedy modularity optimization method under the four experimental conditions. N: healthy mice supplemented with PBS (Figure S1); C: healthy mice supplemented with a daily dose of 150 mg/kg bodyweight of chondroitin sulfate disaccharide (CSD) for 16 consecutive days (Figure S2); M: mice subjected to exhaustive exercise stress supplemented with PBS (Figure S3); S: the stressed mice supplemented with a daily dose of 150 mg/kg bodyweight of CSD for 16 days (Figure S4). The color of each node (OTU) represents the phylum to which the OTU belongs. The circles (dashed lines) indicated the approximate positions of some key modules (subnetworks) in the global network.


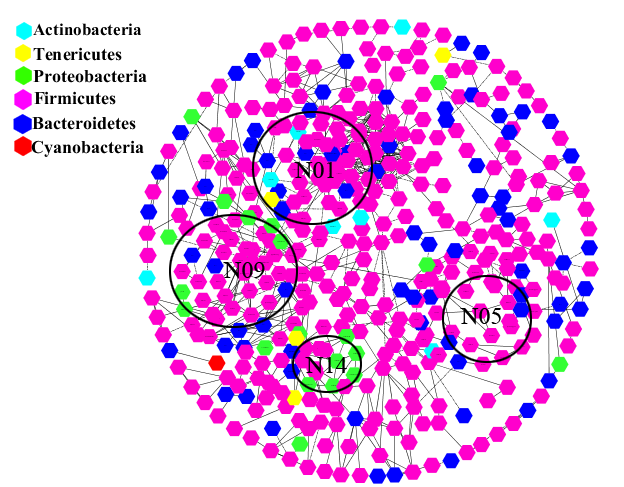


**Figure S1.** Visualization of global networks with modules identified using the fast greedy modularity optimization method under the four experimental conditions in healthy mice supplemented with PBS.


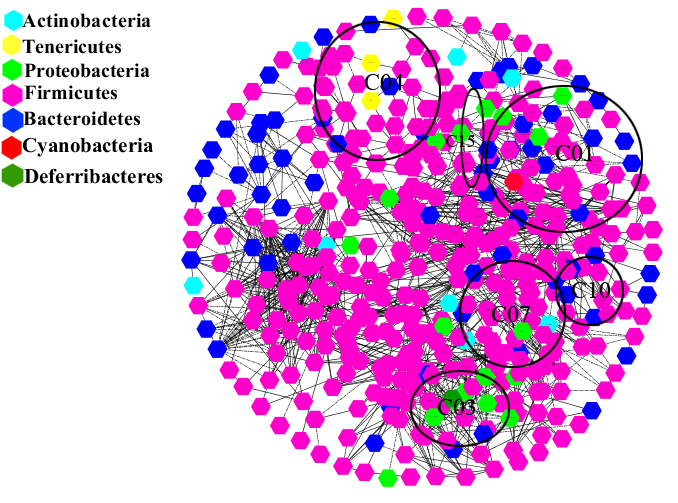


**Figure S2.** Visualization of global networks with modules identified using the fast greedy modularity optimization method under the four experimental conditions in healthy mice supplemented with a daily dose of 150 mg/kg bodyweight of chondroitin sulfate disaccharide (CSD) for 16 consecutive days.


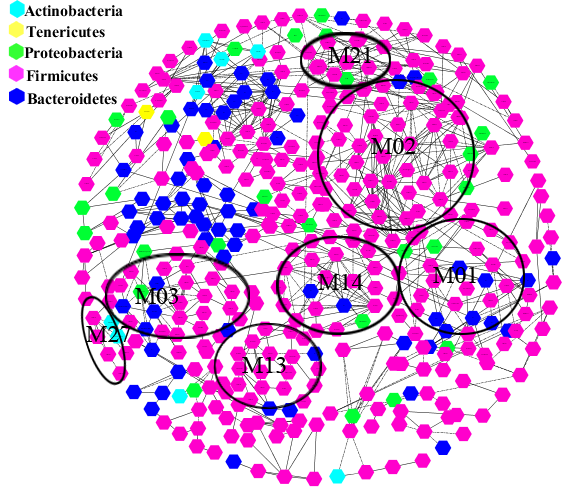


**Figure S3.** Visualization of global networks with modules identified using the fast greedy modularity optimization method under the four experimental conditions in mice subjected to exhaustive exercise stress supplemented with PBS.


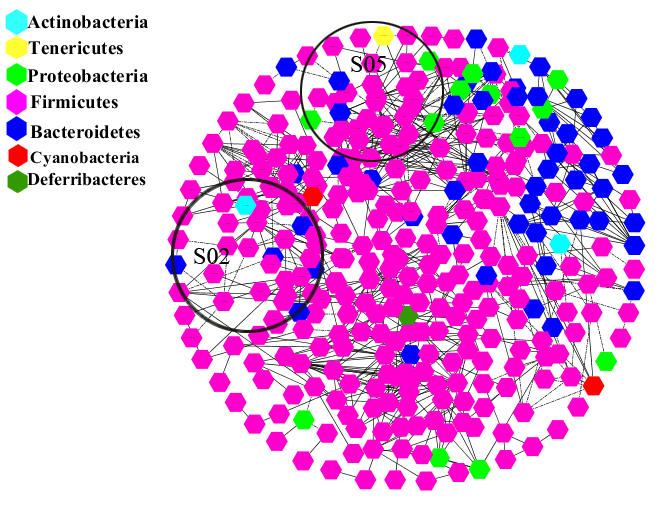


**Figure S4.** Visualization of global networks with modules identified using the fast greedy modularity optimization method under the four experimental conditions in the stressed mice supplemented with a daily dose of 150 mg/kg bodyweight of CSD for 16 days.
